# Perfluoropropionic Acid (CF_3_CF_2_C(O)OH): Three Conformations and Dimer Formation

**DOI:** 10.3390/molecules30091887

**Published:** 2025-04-23

**Authors:** Carlos O. Della Védova, Rosana M. Romano, Hans-Georg Stammler, Norbert W. Mitzel

**Affiliations:** 1 Centro de Química Inorgánica “Dr. Pedro J. Aymonino”, CEQUINOR (Universidad Nacional de La Plata, UNLP, Centro Científico y Tecnológico, Consejo Nacional de Investigaciones Científicas y Técnicas, CCT-CONICET La Plata, Associated with Comisión de Investigaciones Científicas de la Provincia de Buenos Aires, CIC-PBA), Departamento de Química, Facultad de Ciencias Exactas, Universidad Nacional de La Plata, Boulevard 120 N° 1465, La Plata CP 1900, Argentina; carlosdv@quimica.unlp.edu.ar; 2 Lehrstuhl für Anorganische Chemie und Strukturchemie, Center for Molecular Materials CM2, Bielefeld University, Universitätsstraße 25, 33615 Bielefeld, Germany; georg.stammler@uni-bielefeld.de

**Keywords:** perfluoropropionic acid, low-temperature crystal structure, cryogenic Ar matrix study, conformers, monomers, dimer, computational calculations

## Abstract

Perfluoropropionic acid (CF_3_CF_2_C(O)OH) has been investigated with a focus on its complex structural properties. As a formal derivative of propanoic acid, the incorporation of fluorine atoms imparts unique structural features, including three distinct monomeric conformations and a dimeric structure. This study presents experimental findings, supported by computational modeling, to explore these characteristics. The analysis includes an FTIR study of the isolated species in an Ar-cryogenic matrix and the low-temperature determination of its crystalline structure using single-crystal X-ray diffraction.

## 1. Introduction

The chemistry of fluorine has long been, and continues to be, with renewed momentum, one of the most captivating fields for chemists worldwide. Fluorine, in its compounds, cannot be simply regarded as either a “larger hydrogen” or a “smaller chlorine”. The properties of its compounds often prove to be unexpected, unpredictable, fascinating, and truly unique. For example, the energy transition has once again positioned fluorine at the forefront of the discipline. It is a key component of the salt used in lithium battery electrolytes, LiPF_6_, and in its elemental form plays a vital role in chemically eliminating trace water by oxidizing it into OF_2_ and HF [1].

In particular, fluoro- and perfluoro-organic compounds have found a wide range of applications. Specifically, perfluoroorganic compounds, one of which is the focus of this work, exhibit remarkable stability due to the presence of C-F bonds within their molecular structure. This stability—thermal, chemical, biological, and, to some extent, photochemical—confers upon them a significant degree of environmental persistence [2,3,4,5,6,7,8,9,10,11,12,13].

Perfluoropropionic acid, CF_3_CF_2_C(O)OH (PFPA), the title compound, may not accumulate in the environment to the same extent as the longer chain perfluorinated carboxylic acids [7,8], and its natural sources have not been identified so far. That it has been detected in rainwater [14,15,16], however, stresses its role as an environmentally active molecule. To fully understand how this and other long-lived products are formed under the complex environmental conditions present in a given reactive matrix, it is essential to acquire a detailed knowledge of the photochemical evolution of the species, their association equilibria at various temperatures, and to experimentally determine the existence of all conformers present at room temperature.

In the context of the anhydride acid molecule central to this study, it is worth noting that, from a structural perspective, the hydrate and dihydrate of anhydrous acid were investigated using Chirped-Pulse Fourier Transform Microwave (CP-FTMW) Spectroscopy. The study revealed that the complexation of the -OH group of the acid with one or two water molecules occurs on the plane of the carboxylic acid group, resulting in the formation of a six- or eight-membered ring structure [17].

Important for this work is a vibrational study of CF_3_CF_2_C(O)OH, published by Crowder in 1972, in which he detailed the partial and total association of the species in the vapor and liquid states, respectively. The use of fundamental vibrational concepts, such as evaluating group electronegativity, allowed him to understand, for example, the shift to higher wavenumbers of the carbonyl stretch when a CF_3_CF_2_- group is formally replaced by a CF_3_- group, and its connection with the hydrogen bonding comparison between the two species [18,19].

Another study explored the far-infrared spectra of a set of 27 carboxylic acids in aqueous solution, including the compound examined in this work. The analysis of the spectra, particularly in the OH stretching region, enables the determination of conformational isomerism [20]. Computational studies using DFT-B3LYP/6-311+G** and ab initio MP2/6-311+G** calculations on perfluoropropionic acid revealed the existence of an equilibrium between two conformations: the *cis* form (where the C=O group eclipses the C-C bond) and the *gauche* conformer. The calculations predict the *gauche* conformer to be the lower-energy form at ambient temperature, with an abundance of 76% *gauche* and 24% *cis* at 298.15 K [21]. The structural properties of perfluoropropionic acid have been resolved through the study of its rotational spectrum using a pulsed nozzle, chirped-pulse Fourier transform microwave spectrometer within the frequency range of 8–14 GHz. Combined quantum chemical calculations and spectroscopic analysis supports the assignment of the *gauche* form, with a C−C−C=O dihedral angle of 106–107°, and variations depending on the level of approximation used [22].

In another work, the chemistries of perfluoropropionic acid and its close derivatives were studied, described, and compared [23]. Perfluoropropionic acid was also included in an early study utilizing neutron spectroscopy to examine its vibrational spectrum, alongside a broader group of related organic acids [24]. The title compound was also investigated using gas-phase mid-IR, near-IR, and visible vibrational spectroscopy, alongside perfluorooctanoic and perfluorononanoic acid, employing Fourier transform and cavity ring-down spectroscopy. The authors of this work concluded that these compounds exhibit more harmonic O–H bonds, lower transition wavenumbers, and reduced intensities compared to shorter-chain hydrocarbon acids, alcohols, and peroxides [25]. The vibrational spectra of the title compound were also found within the range of 11,000–1000 cm^−1^ and were compared with those of its hydrocarbon homolog, propionic acid [26]. Perfluoropropionic acid was one of the compounds used to study a simple drop-coating deposition using Raman spectroscopy methods to concentrate perfluoroalkyl substances and subsequently design an accessible and reliable spectral library [27].

A family of polyfluorinated compounds, including CF_3_CF_2_C(O)OH, was analyzed from the perspective of the fragmentation process of the deprotonated species. It was demonstrated that the relative energy of the transition state of the formed CCFC ring, which leads to the FCO_2_^−^ anion, is directly linked to the subsequently observed dissociation [28]. In another work, we also employed perfluoropropionic acid to study details related to its photoexcitation, photoionization, and photofragmentation using synchrotron light energies in the range between 11.7 and 715.0 eV. At low energies, the detected fragments were COH^+^, C_2_F_4_^+^, and the parent M^+^ ion. In this work, and in line with the experimental variables used—for instance, very low pressures on the order of 10^−6^ mm Hg—there was no evidence of the existence of a dimer of perfluoropropionic acid [29].

CF_3_CF_2_C(O)OH was also part of a very recent study that evaluated the decomposition products of this family of compounds with the aim of providing more information about the thermal evolution process [30]. In this context, and in connection with this evidence, the degradation of perfluoropropionic acid and related compounds was investigated using an argon plasma under various conditions [31].

A previous analysis of this type reported that after decomposition in an N_2_ atmosphere at temperatures between 200 and 780 °C, the identified products were CF_2_=CF_2_, CF_3_CF_2_H, and CF_3_C(O)F. In an O_2_ atmosphere at below 400 °C, the main product is OCF_2_, accompanied by the inevitable formation of SiF_4_ due to the use of a quartz reactor [32]. From a computational perspective, and to understand transport behavior of relatively stable substances over considerable distances, such as perfluoroalkyl and polyfluoroalkyl compounds, this work aims to comparatively determine the gas-phase thermochemical properties of the compounds, which includes perfluoropropionic acid [33]. The use of perfluoropropionic acid for studying its role in the nucleation of atmospheric molecules under ambient conditions is computationally analyzed in order to understand, at a molecular level, the composition and formation mechanism of secondary organic aerosols [34]. In connection with the above-mentioned decomposition processes, we highlight that pentafluoropropionate salts (salts of Li, Na, K, Cs, Mg, Ca and Ba) were also examined. In that study, the principal pyrolysis product of the pentafluoropropionate salts under dynamic vacuum was tetrafluoroethylene (CF_2_=CF_2_) [35].

To fully comprehend how this and other long-lived products are formed under the intricate environmental conditions present in a given reactive matrix, it is crucial to gain a detailed understanding of the photochemical evolution of the species and their association equilibria at various temperatures and to experimentally confirm the existence of all conformers present at room temperature. This comprehensive approach ensures a deeper insight into the mechanisms and interactions that drive the formation and stability of these products.

## 2. Results and Discussion

### 2.1. Quatum Chemical Calculations

#### 2.1.1. Monomer

With the aim of determining which conformations of CF_3_CF_2_C(O)OH coexist in the gas phase at room temperature, a potential energy surface was calculated as a function of the dihedral angles *φ*(C−C−C=O) and *φ*(O−C−O−H), using the B3LYP/6-311+G(D) approximation (Figure S1).

**Figure 1:**
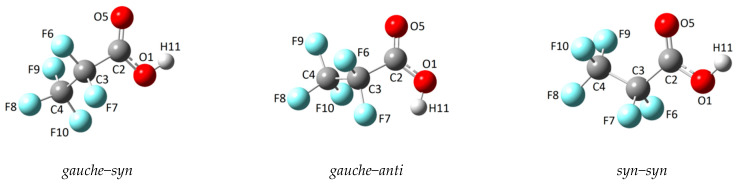
Optimized structures (MP2/6-311+G(D)) of the *gauche*–*syn*, *syn*–*syn*, and *gauche*–*anti* conformers of CF_3_CF_2_C(O)OH.

The *gauche*–*syn*, *gauche*–*anti*, and *syn*–*syn* conformations (Figure 1) correspond to minima on the aforementioned potential energy surface, while the *syn*–*anti* structure corresponds to a saddle point. This represents a notable difference between CF_3_CF_2_C(O)OH and its hydrogenated analog, which admits four stable conformations. Despite the stability of three CF_3_CF_2_C(O)OH conformations, only one (*gauche*–*syn*) has been properly detected experimentally and studied.

The structures corresponding to the three mentioned conformers were optimized and their harmonic vibrational frequencies were calculated using different approximations. Table 1 presents the values of the dihedral angles *φ*(C−C−C=O) and *φ*(O−C−O−H) for the different conformations, their relative energies, and their populations determined using the Boltzmann equation at room temperature, taking into account the double degeneracies for the *gauche* species due to symmetric considerations, using the MP2/6-311+G(D) level of approximation.

The obtained and tabulated results indicate that the *gauche*–*syn* conformer has the highest conformational population percentage at 298 K (85.1%), followed by the *syn*–*syn* rotamer (14.7%) and finally the *gauche*–*anti* form (0.2%).

Table S1 (Supplementary Information) lists the theoretically calculated vibrational wavenumbers for each of the three conformers, obtained at the MP2/6-311+G(d) level of theory, which correspond to the experimental wavenumber range. A tentative spectral assignment is also provided. These data will later facilitate the interpretation of the FTIR spectra of matrix-isolated CF_3_CF_2_C(O)OH.

#### 2.1.2. Dimer

**Figure 2:**
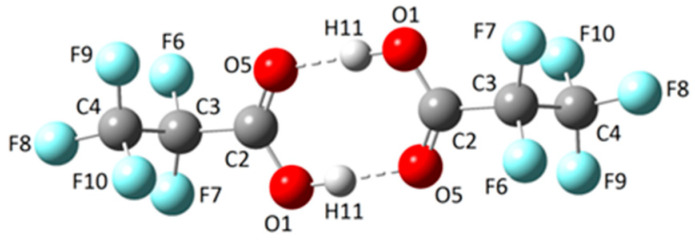
Optimized structures (MP2/6-311+G(D)) of the dimer of CF_3_CF_2_C(O)OH.

The structure of the CF_3_CF_2_C(O)OH dimer (Figure 2) was calculated using the MP2/6-311+G(D) approximation, taking into account that the structure determined by X-ray diffraction reproduces these data (see X-ray diffraction section). The dimer consists of two enantiomeric monomeric units (*gauche*–*syn*) that are properly oriented and linked to each other through two hydrogen bonds. The calculated geometry belongs to the *C*_i_ point group.

The IR spectrum of the dimeric form was computed at the MP2/6-311+G(d) level of theory. The wavenumbers of the IR-active vibrational modes, along with their tentative assignments, are compiled in Table S1. Similarly to the simulated spectra of the monomeric conformers, the theoretical dimer spectrum serves as a key reference for interpreting and assigning the experimental gas-phase and matrix-isolation IR spectra.

### 2.2. Experimental Results

#### 2.2.1. Gas-Phase FTIR Spectra

**Figure 3:**
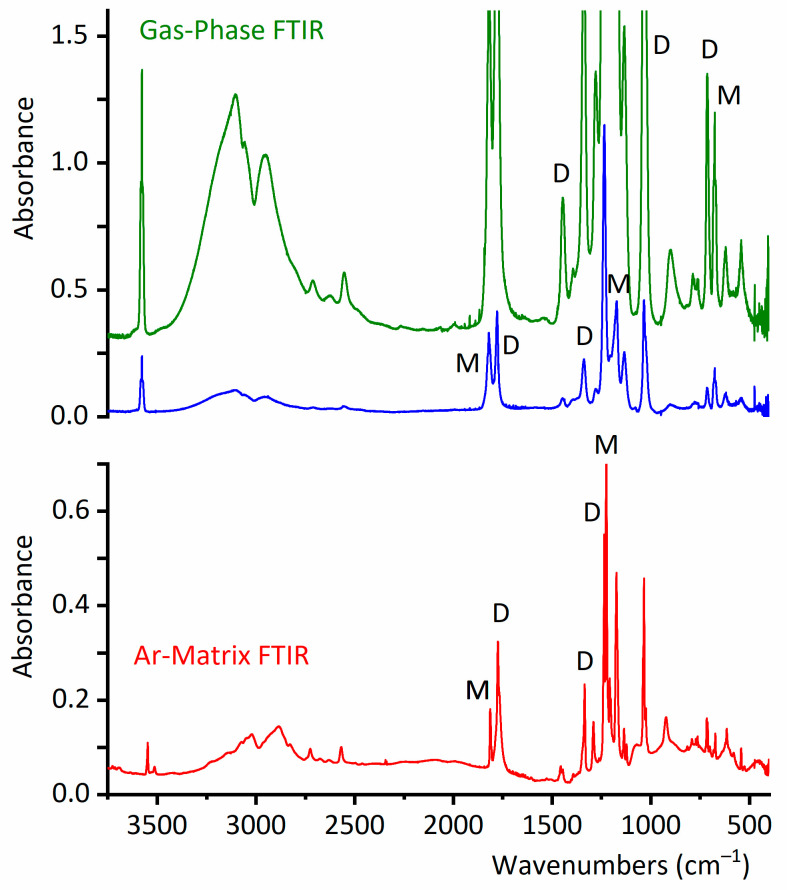
Gas-phase FTIR spectra of CF_3_CF_2_C(O)OH (optical path, 10 cm; resolution, 0.5 cm^−1^; and pressures, 40 torr (top, green line) and 8 torr (middle, blue line)) and the Ar matrix FTIR spectrum (bottom, red line) with a CF_3_CF_2_C(O)OH:Ar ratio at 1:500 and with a resolution of 0.5 cm^−1^, in the 3740–400 cm^−1^ wavenumber region. Some of the bands of the monomer and dimer species are indicated by M and D, respectively.

**Figure 4:**
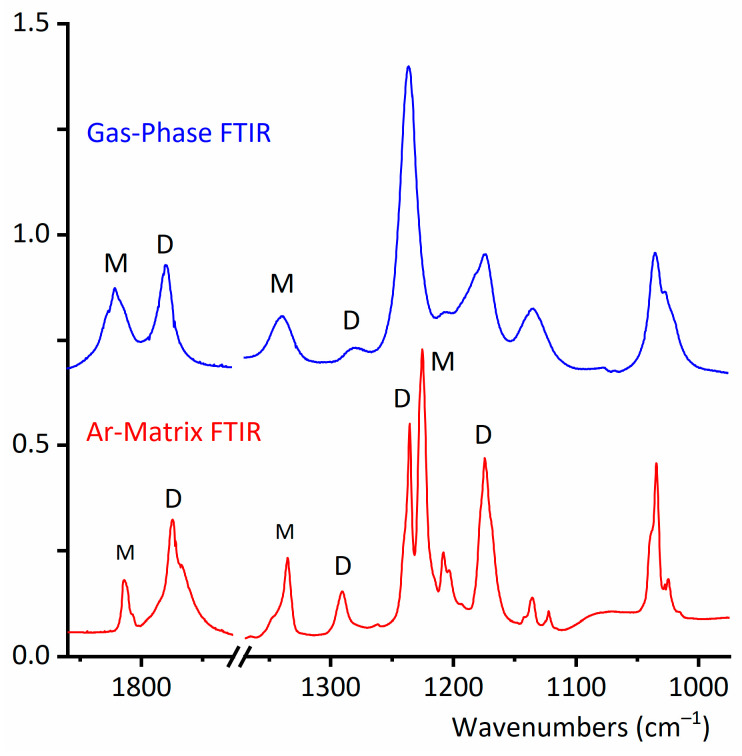
Selected regions of the gas-phase FTIR spectrum of CF_3_CF_2_C(O)OH (optical path, 10 cm; resolution, 0.5 cm^−1^; pressure, 8 torr (top, blue line)) and the Ar matrix FTIR spectrum (bottom, red line) with a CF_3_CF_2_C(O)OH:Ar ratio at 1:500 and a resolution of 0.5 cm^−1^. Some of the bands of the monomer and dimer species are indicated by M and D, respectively.

In 1972, Crowder reported for the first time the infrared spectrum of the gas and liquid phases of CF_3_CF_2_C(O)OH [18]. The experimental gas-phase FTIR spectrum of CF_3_CF_2_C(O)OH demonstrates clear evidence for the simultaneous presence of monomeric and dimeric forms (Figure 3 and Figure 4). Despite the good resolution of the acquired spectra (0.5 cm^−1^), the different conformational contributions of the monomer remain unclear. The gas-phase infrared spectrum assignment is detailed in Table S1.

The gas-phase IR spectra (Figure 3 and Figure 4) exhibit six different absorptions attributable to the dimeric species. Notably, the ν(O−H) stretching vibration undergoes a significant redshift from 3576 cm^−1^ (monomer) to ~3100 cm^−1^ (dimer), consistent with strong hydrogen bonding between subunits. Due to the dimer’s *C*_i_ symmetry, only the antisymmetric (out-of-phase) O−H stretching fundamental mode is IR-active.

Similarly, the carbonyl stretching vibration shifts from 1821 cm^−1^ (monomer) to 1779 cm^−1^ (dimer), indicating substantial intermolecular interaction via the C=O groups. This observation aligns with the formation of the characteristic cyclic structure of carboxylic acid dimers, as predicted computationally (Figure 2). The experimental wavenumber shifts for these modes show excellent agreement with theoretical calculations (Table S1), validating the proposed dimeric structure.

The wavenumbers and tentative assignments of the four additional dimer absorptions observed in the gas-phase IR spectra are detailed in Table S1. Notably, a distinct band at 900 cm^−1^—assigned to the out-of-phase HCO deformation mode of the dimer—appears in a spectral region devoid of monomer absorptions (Table S1). This feature provides strong spectroscopic evidence for dimer formation.

**Figure 5:**
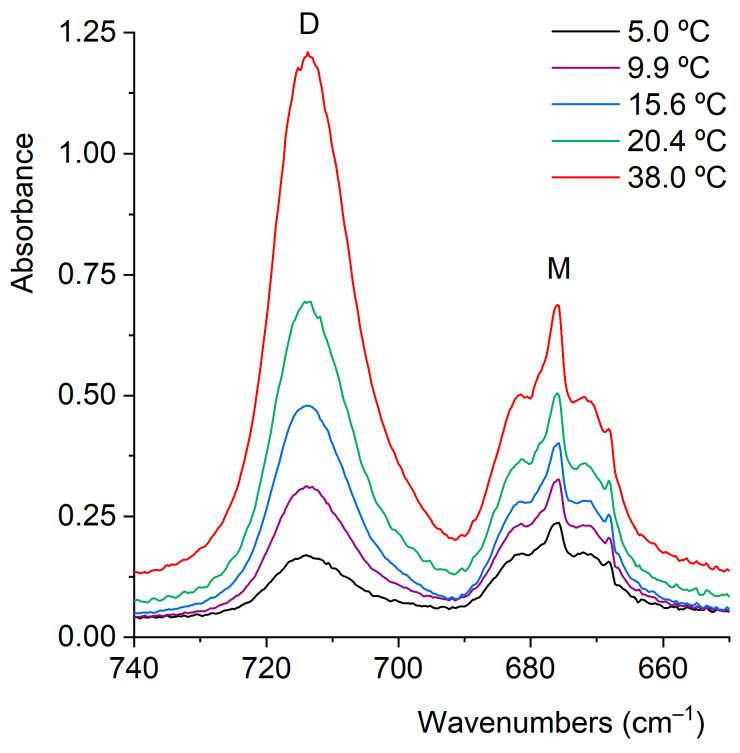
Gas-phase FTIR spectra of CF_3_CF_2_C(O)OH (optical path: 10 cm; resolution: 0.5 cm^−1^) recorded from the liquid phase at different temperatures.

**Figure 6:**
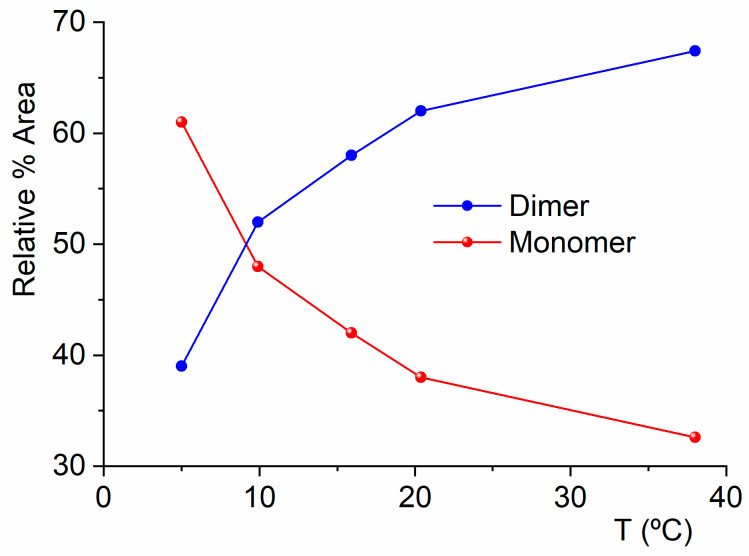
Relative percentage areas of the IR bands at 714 cm^−1^, arising from the bending vibration of the O−C=O group in the dimeric CF_3_CF_2_C(O)OH species, and at 676 cm^−1^, corresponding to the same mode in the monomeric acid in its *gauche*–*syn* conformation, as a function of the liquid temperature in equilibrium with the vapor phase.

Next, the coexistence of monomers and a dimer of CF_3_CF_2_C(O)OH will be confirmed through experiments conducted in the vapor phase. It is important to have this information to ensure that, during the preparation of the CF_3_CF_2_C(O)OH matrix in Ar for deposition and measurement at cryogenic temperature, the dimer concentration is minimized. Figure 5 shows a selected section of the FTIR spectra in the vapor phase for CF_3_CF_2_C(O)OH samples measured at different temperatures. In this region, two distinct bands clearly appear: one at 714 cm^−1^, corresponding to the out-of-phase CF_2_ deformation mode of the dimer, and another at 676 cm^−1^, corresponding to the δ(CF_2_) mode of the monomer. Figure 5 and Figure 6 describe the results of experiments conducted with the vapor phase of the species, aiming to determine the optimal experimental conditions for ensuring that monomeric species predominate over the dimer in the matrix isolation experiments of perfluoropropionic acid. The remaining dimer bands discussed in this section exhibit consistent spectral shifts relative to the monomer absorptions, further supporting their proposed assignments.

According to the experimental design used to record these spectra, the rise in temperature is directly associated with an increase in the vapor pressure of perfluoropropionic acid, promoting monomer interactions and resulting in a higher proportion of dimeric species.

This experiment should not be confused with the one originally conducted by Crowder, who recorded infrared spectra of CF_3_CF_2_C(O)OH at different temperatures while keeping the pressure constant. As expected, the increase in temperature favors the growth of the entropic term associated with the system’s evolution toward the formation of a greater number of monomeric species [18]. These monomeric species, predicted by Crowder, have now been determined with the help of computational calculations.

#### 2.2.2. FTIR Spectrum of CF_3_CF_2_C(O)OH Isolated in Solid Argon

The study of species isolated using cryogenic matrices enhances the resolution of infrared spectra by eliminating the contributions of rotational broadening at low temperatures, typically around 15 K. Thus, the close inspection of the infrared spectrum of the CF_3_CF_2_C(O)OH:Ar (1:500) mixture, in combination with the aid of the computational predictions, reveals the contributions of the *gauche*–*syn* and *syn*–*syn* conformers of CF_3_CF_2_C(O)OH, which coexist in the gas phase. No clear contribution of the less abundant *gauche*–*anti* conformer to the IR spectrum is observed.

Four distinct absorptions assigned to the *syn*–*syn* conformer exhibit measurable wavenumbers shifts relative to the dominant *gauche*–*syn* conformer, with sufficient intensity for detection in the matrix–isolation IR spectrum (Table S1). These bands correspond to ν(C=O) stretching, ν(C_2_-C_3_) stretching, ν(C-O) stretching, and ν_S_(CF_3_) symmetric stretching fundamental modes. The observed shifts align quantitatively with theoretical predictions reported in this study, confirming the spectral assignment of the *syn*–*syn* conformer.

The presence of a second conformation represents a difference between propionic acid, CH_3_CH_2_C(O)OH, and its perfluorinated counterpart: the matrix FTIR spectrum of propionic acid at low temperatures only shows the existence of a single conformer [36]. Figure 4 compares the FTIR spectra of CF_3_CF_2_C(O)OH in the gas phase and in the matrix, highlighting the respective conformational contributions in the latter.

#### 2.2.3. Matrix FTIR Spectra of CF_3_CF_2_C(O)OH After Broadband UV–Vis Irradiation

**Figure 7:**
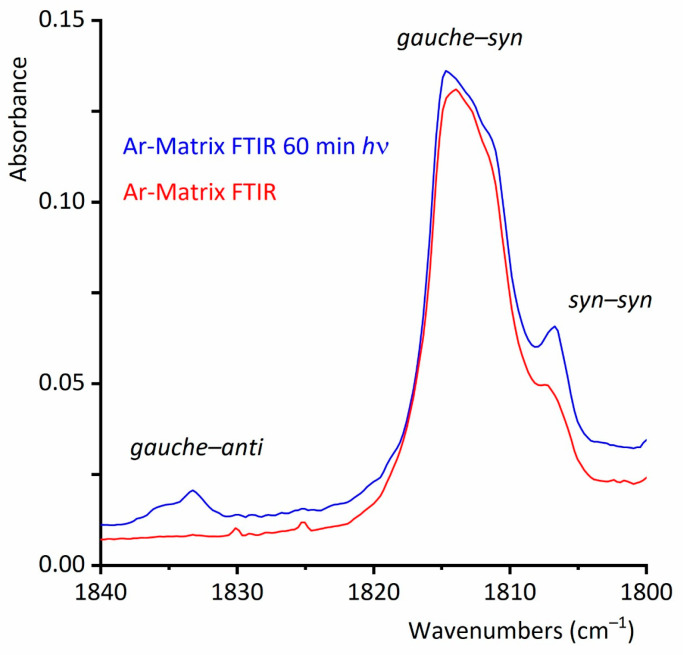
Ar matrix FTIR spectra of CF_3_CF_2_C(O)OH (resolution, 0.5 cm^−1^; CF_3_CF_2_C(O)OH:Ar ratio, 1:500) between 1840 and 1800 cm^−1^, taken immediately after deposition (bottom, red line) and after 60 min of broad band UV–vis irradiation (top, blue line).

The matrix of CF_3_CF_2_C(O)OH diluted in argon in a 1:500 ratio at cryogenic temperatures was exposed to UV–vis broadband irradiation in the range of 200 ≤ *λ* ≤ 800 nm. Spectra were acquired before irradiation and at different irradiation times (0.5, 1.5, 3, 6, 12, 30, and 60 min). The irradiation resulted in a decrease in the population of the lowest energy and most abundant conformer in the gas phase, the *gauche*–*syn* conformer, and an increase in the *syn*–*syn* conformer and the dimeric species. A significant finding was that after 30 min of irradiation, signals of the *gauche*–*anti* form appeared in the spectrum. The ν(O-H) and ν(C=O) vibrational modes of the *syn*–*syn* conformer were observed at higher wavenumbers (3574 and 1834 cm^−1^, respectively) compared to those of the more abundant *gauche*–*syn* conformer. Two additional bands exhibiting similar spectral shifts were detected at 682 and 616 cm^−1^, which were assigned to δ(CF_2_) and δ(CF_3_) deformation modes, respectively. The *gauche*–*syn* conformer had previously been elusive due to its relatively low concentration in the gas phase at room temperature. Thus, we obtained the first experimental evidence for the existence of this conformer. Figure 7 depicts the FTIR spectra of CF_3_CF_2_C(O)OH isolated in Ar, recorded immediately after deposition and after 60 min of broad band irradiation in the carbonyl stretching vibrational region, which is highly sensitive to conformation. For clarity, the spectra were normalized to the carbonyl absorption of the lowest-energy conformer, the *gauche*–*syn* rotamer.

**Figure 8:**
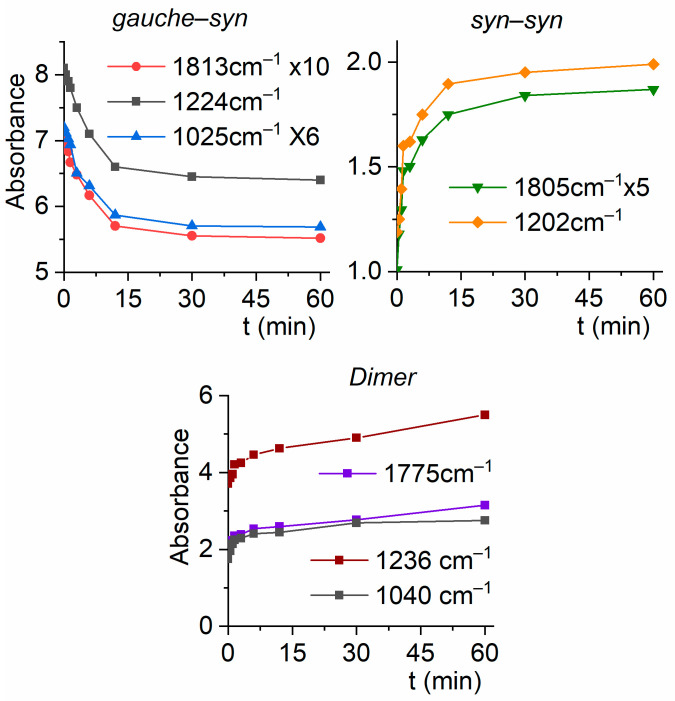
Absorbances of the IR bands of the CF_3_CF_2_C(O)OH:Ar (1:500) matrix for the *gauche*–*syn* (**top left**) and *syn*–*syn* (**top right**) conformers, as well as for the dimer (**bottom**), as a function of irradiation time.

Figure 8 shows the variation in absorbance, measured as the integrated area of the IR bands, as a function of irradiation time. The features assigned to the *syn*–*syn* and *gauche*–*syn* forms increase their intensities at the expense of the bands corresponding to the *gauche*–*syn* rotamer. Additionally, the absorptions of the dimer also increase upon photolysis, presumably due to some monomer diffusion during irradiation.

#### 2.2.4. Solid State Structure

**Figure 9:**
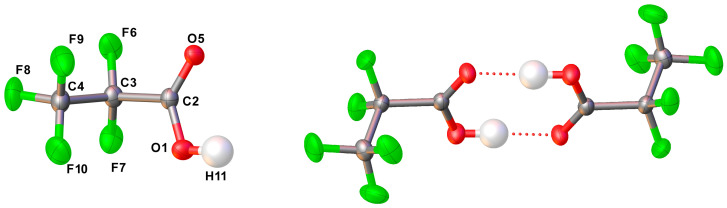
Left: structure of CF_3_CF_2_C(O)OH in the solid state. Thermal ellipsoids are shown at a probability level of 50%. Right: dimer of CF_3_CF_2_C(O)OH; symmetry code used: −*x*, 1 − *y*, −*z*.

The solid-state structure of CF_3_CF_2_C(O)OH has been studied from an in situ grown crystal. CF_3_CF_2_C(O)OH crystallizes in the space group *P*2_1_/*c*, forming dimers in which both monomers adopt a *gauche*–*syn* conformation, related to each other by a crystallographic center of inversion (Figure 9). Table S2 presents crystallographic information obtained from the structural analysis and refinement of CF_3_CF_2_C(O)OH.

The geometric parameters of CF_3_CF_2_C(O)OH obtained by X-ray diffraction are listed below in Table 2, where they are compared with the values obtained by quantum chemistry calculations showing that the calculated values reproduce the experimentally obtained fairly well, even though the computational values would be closer to those determined in the gas phase due to their intrinsic nature.

According to NBO quantum chemical calculations at the B3LYP/6-311+G(D) level of the theory, the aforementioned hydrogen bonds, which are responsible for dimer formation, arise from electronic transfer from a lone pair of the carbonyl oxygen to the anti-bonding molecular orbital σ*(O−H). This interaction leads to a second-order perturbation stabilization energy *E*^(2)^ of 9.20 kcal mol^−1^.

**Figure 10:**
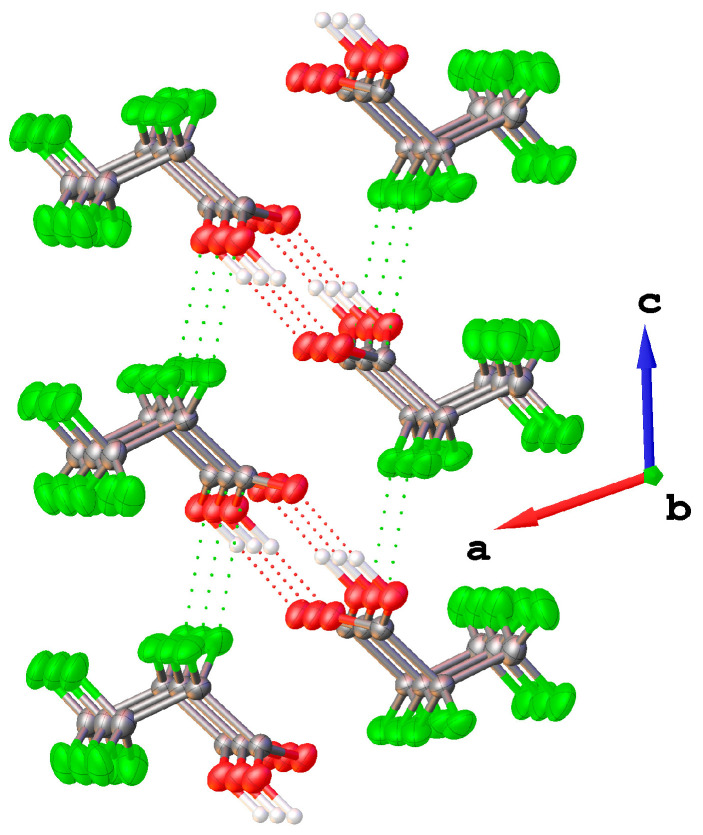
Section of the crystal lattice. Intermolecular contacts below the van der Waals distances are shown as dotted lines.

The hydrogen bond H11···O5′ (symmetry code −*x*, 1 − *y*, −*z*) has a length of 1.70(3) Å, and the distance O1···O5′ is 2.665(2) Å. Additionally, the only appreciable distance below the van der Waals distance (*r*_vdW_) [37] is the contact F6···C2′ (symmetry code: +*x*, 3/2 − *y*, 1/2 + *z*) with 3.113(2) Å. However, the stabilization energy calculated for these interactions is not appreciable. All intermolecular F···O and F···F distances are longer than their van der Waals distances. A section of the crystal lattice, viewing roughly along the *b* axis, is shown in Figure 10.

## 3. Materials and Methods

### 3.1. CF_3_CF_2_C(O)OH

The CF_3_CF_2_C(O)OH 97% was purchased from Sigma Aldrich (Saint Louis, MO, USA) and subsequently purified by distillation through a series of U-shaped cold traps immersed in cold baths at −50, −80, and −110 °C, respectively. The acid was collected in the trap cooled to −50 °C.

### 3.2. Quantum Chemical Calculations

The Gaussian 03 program [38] was used to perform quantum chemical calculations, including the calculation of the potential energy function for a specific dihedral angle, followed by the geometry optimization of the corresponding minima and the calculation of their harmonic wavenumbers. For these purposes, the DFT [39] and MP2 [40] methods were chosen in conjunction with the 6-311+G(D) basis set. NBO [41] calculations were performed with the NBO 5.G package [42] incorporated in Gaussian 03. Additionally, the dimeric structure was computed using Gaussian 03. In this case, a potential energy curve was generated, followed by the optimization of the obtained minimum, employing the B3LYP/6-311+G(D) level of approximation.

### 3.3. Infrared Spectroscopy

Infrared spectra were recorded using a Nicolet™ 6700 spectrometer (Thermo Electron Corporation, Madison, WI, USA) with a double-wall cell featuring a 10 cm optical path length and 0.5 mm thick Si windows. The spectral resolution was 0.5 cm^−1^, and each spectrum was obtained by averaging 64 scans. To optimize the equilibrium between monomers and dimers of perfluoropropionic acid in the matrix study at cryogenic temperatures, spectra were recorded at various temperatures (5.0, 9.9, 15.6, 20.4, and 38.0 °C) to determine the best experimental conditions.

### 3.4. Matrix Isolation Experiments

The gas mixture was deposited on a 15 K CsI window using the pulse deposition technique [43,44,45]. Low temperatures were achieved using a Displex closed-cycle refrigerator SHI-APD Cryogenics, model DE-202(AS Scientific Products, Abingdon, UK). The corresponding FTIR spectra were acquired with the previously described instrument. A Spectra-Physics Hg-Xe arc lamp operating at 1000 W was used to irradiate the matrix within the 200–800 nm broad band range. To prevent matrix heating, a water filter was placed between the lamp and the matrix. Several spectra were recorded at different irradiation times.

### 3.5. X-Ray Diffraction Analysis

A single crystal of CF_3_CF_2_C(O)OH was grown in situ within a capillary. The sample was filled into a capillary, cooled with liquid nitrogen forming a polycrystalline material. At 180 K, a solid/liquid equilibrium near the melting point was established by melting the solid, leaving only a tiny crystal seed intact, using a thin copper wire as an external heat source. The temperature was then gradually lowered at 1 K/h to 176 K, at which point the entire capillary was filled with the crystalline specimen, followed by cooling to 146 K at a rate of 44 K per hour.

The crystal was maintained at 146.0(1) K during data collection, which was performed using an Agilent SuperNova diffractometer (Agilent, Santa Clara, CA, USA) at Bielefeld University. Using Olex2 [46], the structure was solved by direct methods with SHELX-97 [47], and the refinement was carried out using Olex2.refine [48] and spherical scattering factors were calculated with NoSpherA2 [49].

## 4. Conclusions

The evaluation of the results obtained through various spectroscopic techniques—vibrational IR, matrix IR spectra with UV–vis broad band irradiation, and X-ray diffraction analysis—provide complementary data that allow for the study of conformations and equilibria in different families. It is worth noting that conformational properties are crucial for understanding the chemical behavior of macromolecules and are responsible for the fundamental chemical behavior of biological molecules [50,51].

In the present case, and in relation to the above-mentioned data, the structure of CF_3_CF_2_C(O)OH, its dimer, and the existence of three conformations in equilibrium in the vapor phase have been conclusively determined. The combined analysis of matrix–isolation IR spectra and computationally predicted vibrational wavenumbers provides a powerful approach for identifying rotamers and investigating conformational equilibria. This is particularly evident when monitoring IR spectral changes induced by irradiation, which serve as distinctive markers for different conformers.

It is worth noting that the accurate assessment of the number of real conformers is a key tool for precisely approximating the electronic spectra of molecules. A purely computational approach requires evaluating thousands of geometries. In a recently published study, this computational effort is drastically reduced by using effective conformers for the calculation of UV–vis spectra [52].

### Dedication

With all our gratitude, deepest affection, and boundless admiration, we dedicate this work to Prof. Jaan Laane. His unwavering devotion to knowledge—pursued with quiet resilience and constant passion—has been a shining inspiration in our lives. Beyond his brilliance as a scientist and mentor, it is his kindness, his generosity of spirit, and the profound care he has shown us that we will carry with us always. From the unforgettable days in College Station to the laughter-filled, sunny moments in La Plata, every memory made with him has been a gift.

## Figures and Tables

**Table 1:** Energy and Gibbs free energy differences among *gauche*–*syn*, *gauche*–*anti*, and *syn*–*syn* conformers of CF_3_CF_2_C(O)OH; dihedral angles *φ*(C−C−C=O)) and *φ*(O−C−O−H); and conformational population *χ* calculated at 298 K using the MP2/6-311+G(D) approximation.

Conformer	*φ*(C−C−C=O)	*φ*(O−C−O−H)	Δ*E* (kcal/mol)	Δ*G* (kcal/mol)	*χ* (%)
*gauche*–*syn*	101.2	−0.3	0.00	0.00	85.1
*syn*–*syn*	−0.1	0.0	0.43	0.62	14.7
*gauche*–*anti*	82.3	176.6	3.37	3.64	0.2

**Table 2:** Experimental structure parameters obtained by X-ray diffraction and computed parameters (distances in Å; angles in degrees) corresponding to the *gauche*–*syn* conformer of CF_3_CF_2_C(O)OH.

Parameter	X-Ray Diffraction	MP2/6-311+G(D)
*r*(F6−C3)	1.338(2)	1.344
*r*(F7−C3)	1.336(2)	1.351
*r*(F9−C4)	1.319(2)	1.336
*r*(F8−C4)	1.317(2)	1.330
*r*(F10−C4)	1.304(2)	1.332
*r*(O5=C2)	1.215(2)	1.203
*r*(O1−H11)	0.97(3)	0.971
*r*(O1−C2)	1.286(2)	1.337
*r*(C2−C3)	1.545(2)	1.542
*r*(C4−C3)	1.542(2)	1.542
*α*(H11−O1−C2)	112.6(17)	108.3
*α*(O5−C2−O1)	127.9(2)	126.9
*α*(O5−C2−C3)	120.0(2)	123.0
*α*(O1−C2−C3)	112.0(2)	110.0
*α*(F9−C4−F8)	108.9(2)	109.0
*α*(F9−C4−F10)	108.8(2)	108.8
*α*(F9−C4−C3)	109.4(2)	109.4
*α*(F8−C4−F10)	109.1(2)	108.9
*α*(F8−C4−C3)	110.1(2)	110.1
*α*(F10−C4−C3)	110.6(2)	110.5
*α*(F6−C3−C7)	108.7(2)	108.9
*α*(F6−C3−C2)	109.0(2)	108.8
*α*(F6−C3−C4)	108.1(2)	107.7
*α*(F7−C3−C2)	110.5(2)	110.6
*α*(F7−C3−C4)	107.9(2)	108.0
*α*(C2−C3−C4)	112.6(2)	112.8
*τ*(H11−O1−C2−O5)	−0.5(18)	−0.3
*τ*(H11−O1−C2−C3)	−178.2(17)	178.8
*τ*(O5−C2−C3−F6)	21.2(2)	−18.2
*τ*(O5−C2−C3−F7)	140.6(2)	−137.8
*τ*(O5−C2−C3−C4)	−98.7(2)	101.2
*τ*(O1−C2−C3−F6)	−160.9(2)	162.6
*τ*(O1−C2−C3−F7)	−41.5(2)	43.1
*τ*(O1−C2−C3−C4)	79.2(2)	−78.0
*τ*(F9−C4−C3−F6)	−65.5(2)	65.1
*τ*(F9−C4−C3−F7)	177.2(2)	−177.4
*τ*(F9−C4−C3−C2)	54.9(2)	−54.9
*τ*(F8−C4−C3−F6)	54.1(2)	−54.7
*τ*(F8−C4−C3−F7)	−63.3(2)	62.8
*τ*(F8−C4−C3−C2)	174.5(2)	−174.7
*τ*(F10−C4−C3−F6)	174.7(2)	−175.0
*τ*(F10−C4−C3−F7)	57.3(2)	−57.6
*τ*(F10−C4−C3−C2)	−64.9(2)	65.0

## Data Availability

The original contributions presented in this study are included in the article/Supplementary Materials. Further inquiries can be directed to the corresponding author.

## Supplementary Materials

The following supporting information can be downloaded at: https://www.mdpi.com/article/10.3390/molecules30091887/s1. Figure S1: Potential energy surface of CF_3_CF_2_C(O)OH as a function of the dihedral angles *φ*(O−C−O−H) and *φ*(C−C−C=O), displayed from different perspectives; Table S1: Comparison between experimental and computed IR vibrational modes for CF_3_CF_2_C(O)OH and their proposed assignment; Table S2: Crystallographic information for CF_3_CF_2_C(O)OH.
