# Supplementary material for: Perfluoropropionic Acid (CF3CF2C(O)OH): Three Conformations and Dimer Formation
Source: Molecules. 2025 Apr 23;30(9):1887. doi: 10.3390/molecules30091887 (PMC12073299; doi:10.3390/molecules30091887)
Supplement: Supplementary file 1 [file molecules-30-01887-s001.zip › molecules-3557904-supplementary.pdf]

Supplementary Material

Perfluoropropionic acid ($CF_3CF_2C(O)OH$): Three conformations and dimer formation

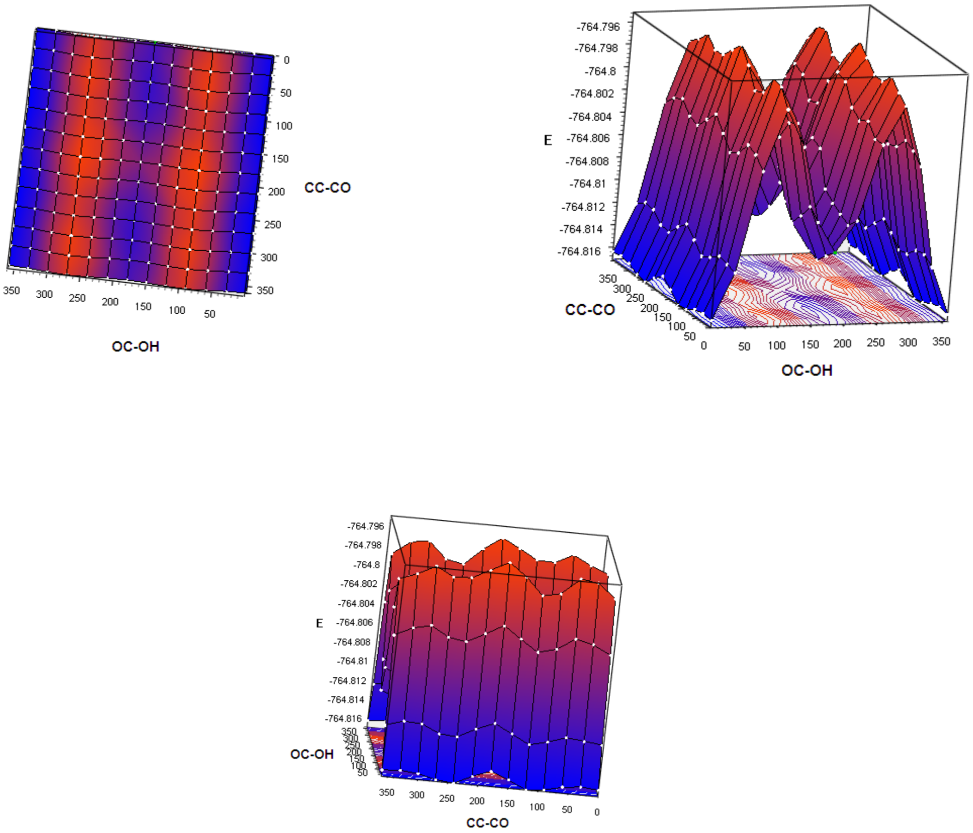

Figure S1. Potential energy surface of $CF_3CF_2C(O)OH$ as a function of the dihedral angles φ(O−C−O−H) and φ(C−C−C=O), displayed from different perspectives.

Table S1. Comparison between experimental and computed IR vibrational modes for $CF_3CF_2C(O)OH$ and their proposed assignment.

Experimental				MP2/6-311+G(D)[a]								Proposed assignament
Gas Phase		Matrix $CF_3CF_2C(O)OH$:Ar (1:500)		*gauche-syn*		*syn-syn*		*gauche-anti*		Dimer[b]		
ν / cm^{-1}	Int.[c]	ν / cm^{-1}	Int.	ν / cm^{-1}	Int.	ν / cm^{-1}	Int.	ν / cm^{-1}	Int.	ν / cm^{-1}	Int.	
3576	0.204	3574						3586	<1			ν(O–H)
		3547	0.113	3556	23	3556	4					
3107	0.087	3228	0.083							3276	100	$(\nu(O–H)_{o.o.p.})_{dimer}$
1821	0.284	1834						1767	<1			ν(C=O)
		1813	0.187	1751	66							
		1805	0.076			1745	11					
1779	0.355	1775	0.393							1732	32	$(\nu(C=O)_{o.o.p.})_{dimer}$
1446	0.056	1457/1447	0.100							1411	5	$(\nu(C2–C3)_{o.o.p.})_{dimer}$
1395	0.055	1403/1393	0.072			1359	4					ν(C2–C3)
		1378/1372/1366	0.130	1351	13							
								1326	<1			
1339	0.192	1335	0.307			1338	2					ν(C3–C4)
				1307	23					1306	11	
								1302	<1			
1279	0.091	1290	0.197							1245	10	$(\nu_{as}(C4F_3)_{o.o.p.})_{dimer}$
1235	1.000	1240	0.372					1201	<1			$\nu_{as}(C4F_3)$
				1197	20							
						1192	15					
		1236	0.752							1189	16	$(\nu_{as}(C4F_3)_{o.o.p.})_{dimer}$
		1224	1.000					1188	<1			$\nu_{as}(C4F_3)$

Experimental				MP2/6-311+G(D)[a]								Proposed assignement
Gas Phase		Matrix $CF_3CF_2C(O)OH$:Ar (1:500)		*gauche-syn*		*syn-syn*		*gauche-anti*		Dimer [b]		
ν / cm^{-1}	Int.[c]	ν / cm^{-1}	Int.	ν / cm^{-1}	Int.	ν / cm^{-1}	Int.	ν / cm^{-1}	Int.	ν / cm^{-1}	Int.	
				1183	85							
						1181	5					
										1182	40	$(\nu_{as}(C3F_2)_{o.o.p.})_{dimer}$
1205	0.205	1208	0.323	1173	38							$\nu_{as}(C3F_2)$
								1168	<1			
						1167	10					
		1203	0.263							1162	4	$(\nu_s(C3F_2)_{o.o.p.})_{dimer}$
1173	0.391	1193	0.15					1153	<1			$\nu_s(C3F_2)$
				1144	44							
						1121	7					
		1174	0.634							1136	26	$(\nu_s(C4F_3)_{o.o.p.})_{dimer}$
1134	0.217	1142	0.109			1115	4					ν(C−O)
		1136/1122	0.308	1108	61							
								1086	<1			
1034	0.397	1040	0.363							1002	17	$(\nu(C-O)_{o.o.p.})_{dimer}$
		1035	0.604	996	35							$\nu_s(C4F_3)$
		1019	0.11			994	13					
								989	<1			
900	0.037	922	0.186							827	12	$(\delta(HCO)_{o.o.p.})_{dimer}$
775	0.044					754	1					$\delta_{o.o.p.}(C=O)$
		791	0.116							752	1	
		771	0.112	745	6							

Experimental				MP2/6-311+G(D)[a]								
Gas Phase		Matrix $CF_3CF_2C(O)OH$:Ar (1:500)		*gauche-syn*		*syn-syn*		*gauche-anti*		Dimer [b]		Proposed assignament
ν / cm^{-1}	Int.[c]	ν / cm^{-1}	Int.	ν / cm^{-1}	Int.	ν / cm^{-1}	Int.	ν / cm^{-1}	Int.	ν / cm^{-1}	Int.	
								739	<1			
		763	0.119	735	<1	737	<1					δ(HCO)
								730	<1			
										728	2	$(\delta_{o.o.p.}(C{=}O)_{o.o.p.})_{dimer}$
714	0.094	717/712/701	0.410							675	5	$(\delta(C3F_2)_{o.o.p.})_{dimer}$
676	0.163	682	0.079					667	<1			$\delta(C3F_2)$
		673	0.130			653	5					
				651	26							
621	0.071					631	<1					$\delta(C4F_3)$
		622	0.102	610	24							
		616	0.141					586	<1	590	<1	
580	0.034	579	0.064			580	2					$\delta(C4F_3)$
				584	1							
								562	<1			
543	0.059	542	0.082							554	1	δ(CCO)
						547	3					
				527	4							
								519	<1	519	1	
		525	0.031	518	3							δ(CCC)
						514	<1	494	<1	504	1	

[a]Corrected with a scaling factor of 0.95. [b]Only the IR active modes are listed. [c]Intensities of the IR spectrum taken at a pressure of 8 torr.

Table S2. Crystallographic information for $CF_3CF_2C(O)OH$.

	$C_3HF_5O_2$
Formula weight	164.032
Temperature/K	156.0(1)
Crystal system	monoclinic
Space group	$P2_1/c$
a/Å	10.9411(16)
b/Å	5.0964(6)
c/Å	9.7342(13)
β/°	107.431(14)
Volume/Å^3	517.85(13)
Z	4
ρ_{calc}g/cm^3	2.104
μ/mm^{-1}	0.276
F(000)	320.5
Crystal size/mm^3	0.3 × 0.13 × 0.13
Radiation	Mo Kα (λ = 0.71073)
2Θ range for data collection/°	7.8 to 50.08
Index ranges	$-12 \le h \le 13$, $-5 \le k \le 6$, $-10 \le l \le 11$
Reflections collected	2583
Independent reflections	902 [R_{int} = 0.0311, R_{sigma} = 0.0368]
Data/restraints/parameters	902/0/95
Goodness-of-fit on F^2	1.044
Final R indexes [I>=2σ (I)]	R_1 = 0.0306, wR_2 = 0.0605
Final R indexes [all data]	R_1 = 0.0401, wR_2 = 0.0654
Largest diff. peak/hole / e Å^{-3}	0.33/−0.21

CCDC 1471202 contains the supplementary crystallographic data for this paper. These data can be obtained free of charge from The Cambridge Crystallographic Data Centre via https://www.ccdc.cam.ac.uk/structures/.
